# Floral diversity in desert ecosystems: Comparing field sampling to image analyses in assessing species cover

**DOI:** 10.1186/1472-6785-13-22

**Published:** 2013-06-10

**Authors:** Taoufik S Ksiksi, Ali A El-Keblawy

**Affiliations:** 1 Department of Biology, UAE University, Al-Ain, United Arab Emirates; 2 Department of Applied Biology, University of Sharjah, Sharjah, United Arab Emirates

## Abstract

**Background:**

Developing a quick and reliable technique to estimate floral cover in deserts will assist in monitoring and management. The present attempt was to estimate plant cover in the UAE desert using both digital photography and field sampling. Digital photographs were correlated with field data to estimate floral cover in moderately (Al-Maha) and heavily (DDCR) grazed areas. The Kruskal-Wallis test was also used to assess compatibility between the two techniques within and across grazing intensities and soil substrates.

**Results:**

Results showed that photographs could be a reliable technique within the sand dune substrate under moderate grazing (r = 0.69). The results were very poorly correlated (r =−0.24) or even inversely proportional (r =−0.48) when performed within DDCR. Overall, Chi-square values for Al-Maha and DDCR were not significant at P > 0.05, indicating similarities between the two methods. At the soil type level, the Kruskal-Wallis analysis was not significant (P > 0.05), except for gravel plains (P < 0.05). Across grazing intensities and soil substrates, the two techniques were in agreement in ranking most plant species, except for *Lycium shawii*.

**Conclusions:**

Consequently, the present study has proven that digital photography could not be used reliably to asses floral cover, while further testing is required to support such claim. An image-based sampling approach of plant cover at the species level, across different grazing and substrate variations in desert ecosystems, has its uses, but results are to be cautiously interpreted.

## Background

A reliable technique in monitoring species diversity in most ecosystems, especially deserts, has generated much debates and discussions among stake holders [1,2]. A large body of literature agree on the importance of continuous monitoring and assessment of biodiversity, both floral and faunal, especially in deserts. One important constraint in sampling deserts is the need for large number of samples for any data to be representative. Regrettably two major constraints - time and resources - hinder gathering large biodiversity datasets that are useful and reliable [3]. Large field surveys typically have labor as well as time constraints [4].

This is particularly more valid for desert ecosystems, where vegetation is scarce and larger areas are to be sampled for any data to be robust and useful. Logistic constraints in collecting plant data have resulted in limited sampling [5]. Even though, field vegetation sampling offers a practical, rapid and objective method to sample vegetation on large as well as small areas [6]. Additionally, trends in vegetation measurements are difficult to quantify and easy to misinterpret. This was true even for simple field techniques such as the Braun-Blanquet cover-abundance [7]. Results from field studies would be difficult to interpret as there is no exact test available to ensure accuracy and reliability of the collected data [8]. One main reason for the misinterpretation is the limited sampling points that could be surveyed [3]. The way out from these barriers has to certainly involve the new technologies in PC and software developments [9].

Image processing packages offer a solution because biodiversity monitoring and assessment using the conventional field techniques are time consuming and require large funds. Attempts in the past have led to bias in data collection because of limitations in time and funds.

Many reports highlight the bias toward annual species composition rather than perennial ones in deserts [10]. This makes monitoring more difficult if it was not done on the right season of the year. One of the most challenging hurdles facing desert ecosystems, however, is the agreement on a simple and uniform monitoring scheme. Such a scheme has to be quick, low cost, and most importantly reflects any temporal changes on species composition. Generally, monitoring programs would lead to identifying important floral species [11,12]. It is therefore important to use sampling techniques that provide unbiased estimations of vegetation in order to avoid unwanted implications [13,14].

Image analyses provide more information than any other measurement over time [15]. Fortunately, image analyses techniques to classify vegetation have received much attention over the years [16]. While image classification poses a challenge because of the complexity in study areas [17], it is important to properly select the remotely sensed data and the appropriateness of the image analysis technique adopted [18]. Expensive images are in many cases necessary [19], it is believed that, especially with the current advances in imaging technology, over the counter cameras could be used to monitor various variables in desert ecosystems. Vegetation indices from remotely sensed data provide an important component of image classification which deals with temporal variations [15]. Ecosystem variables such as ground cover are key indicators but are also labor intensive and time consuming [20].

With the advent of powerful computer processors and robust digital image analyses softwares, large datasets - using digital cameras - could be generated with minimal time and resource use. But the importance of expert knowledge of lens calibration of photogrammetric equipment cannot be underestimated [21]. Close-range photography has been successfully used in arid lands of the USA [16,22] and in Australia [5]. The set-ups include, one way or another, the use of cameras vertically positioned above the sampled quadrats. Heights of camera ranged from 1.8 meter above ground, covering a one square meter area, to 3.5 meters - covering areas as large as 14 square meters [22].

Unfortunately, little information is available on the use of close range photography, using digital cameras, to assess plant cover within the desert rangelands of the Arabian Gulf. Moreover, the agreements between such remotely sensed data with field assessments have received little attention.

The present study will consequently evaluate the use of digital cameras in estimating plant cover in the United Arab Emirates’ desert ecosystems. More specifically, the aim was to compare results from field sampling of species cover to remotely-sensed data of the same quadrats in a desert area of the UAE.

## Results

### Species ranking within both Al-Maha and DDCR

The correlation coefficient between ranking based on digital photography and field sampling was 0.69 within the sand dune substrate (data not shown). *Leptadenia pyrotechnica*, *colatropis procera* and *cyperus conglomeratus* were ranked first, second and sixth in both methods; respectively (Table 1). The correlation coefficients for sand flats and gravel plains were negative (-0.24 and -0.60; respectively), indicating a very poor negative relationship between digital photography and field sampling of floral cover.

**Table 1:** Ranking of the top 3 plant species using close range photography and field based ranking in a UAE desert ecosystem

**AL-MAHA**	**Scientific names**	**Field-based**	**Image-based**
		**ranking**	**ranking**
Sand dunes	*Leptadenia pyrotechnica*	1	1
	*Crotalaria aegyptiaca*	2	2
	*Calotropis procera*	3	0
Sand flats	*Crotalaria aegyptiaca*	1	2
	*Leptadenia pyrotechnica*	2	1
	*Calotropis procera*	3	0
Gravel plains	*Heliotropium kotschyi*	1	4
	*Leptadenia pyrotechnica*	2	3
	*Rhanterium epapposum*	3	6
**DDCR**			
Sand dunes	*Haloxylon salicornicum*	1	3
	*Leptadenia pyrotechnica*	2	1
	*Cyperus conglomeratus*	3	6
Sand flats	*Calotropis procera*	1	3
	*Leptadenia pyrotechnica*	2	1
	*Haloxylon salicornicum*	3	6
Gravel plains	*Haloxylon salicornicum*	1	4
	*Leptadenia pyrotechnica*	2	1
	*Calotropis procera*	3	3

For the field sampling method, *Leptadenia pyrotechnica* was ranked numbers one or two in all the substrates of Al-Maha, which is partially protected from grazing. However, according to image technique, this species was ranked number one in both sand dunes and sand flats, but not recorded at all on gravel plains. *Calotropis procera* was among the top two species in both sand dunes and sand flats, according to ground methods, but was not recorded in sand dunes according to the image technique. In the gravel plains, two of the top six species of the two techniques (*Heliotropium kotschyi* and *Rhanterium epapposum*) did not appear at all in the other substrate types (sand dunes and sand flats).

The correlation coefficient between ranking based on digital photography and field sampling was 0.24, -0.24 and -0.48 within the sand dunes, sand flats and gravel plains; respectively (Data not shown). *L.pyrotechnica*, was ranked second and first using field and digital photography methods; respectively (Table 1). While *D. glaucum*, a preferred grazing species especially for houbara bustard or *Chlamydotis undulata*[23], was ranked ninth using the field sampling method but was not even listed using digital photography. This highlights the need to complement photography with other techniques when sampling floral cover in desert ecosystems. The homogeneous distribution pattern observed on the sand dunes would be the reason for the positive correlations observed in DDCR of that substrate. However, the clumped pattern observed on both sand flats and gravel plains would explain the lack or the negative correlations observed between the field and photography ranking methods.

According to the ground field method, *H. salicornicum* and *L. pyrotechnica* were ranked among the top three species in the three substrate types, together with *C. conglomeratus* on the sand dunes and *C. procera* in the sand flats and gravel plains. These four species were also among the top six species in the image technique.

### Kruskal-Wallis test results across soil substrates and grazing intensities

Table 2 presents ranking results of the Kruskal-Wallis significance, for specific plant species. Significant Chi-square values (P < 0.05) reveal incompatibilities between field assessment and digital imaging in species ranking. While P levels higher than 0.05 show agreements between the two methods.

**Table 2:** Kruskal-Wallis test results of level of agreements between field and digital imaging, across soil substrates and grazing intensities, in estimating plant cover in a desert ecosystem

**Plant Species**	**Chi-Square values**	**P values**
*Calotropis procera*	0.73	0.394
*Cyperus conglomeratus*	0.69	0.405
*Dipterygium glaucum*	3.43	0.064
*Heliotropium digynum*	0.62	0.431
*Indigofera coultea*	0.91	0.340
*Leptadenia pyrotechnica*	2.86	0.09
*Limeum arabicum*	0.52	0.471
*Lycium shawii*	7.03	0.008
*Milkiopsis ciliata*	0.07	0.790
*Rhanterium epapposum*	0.89	0.346

Both methods had close agreements in ranking all plant species, except for *Lycium shawii*, a thorny shrub sometimes associated with gravel plains [24], at P > 0.05. The Chi-square was 7.03, 3.43 and 2.86 for *Lycium shawii* with P = 0.008, *Dipterygium glaucum* with P = 0.064 and *Leptadenia pyrotechnica* with P = 0.09; respectively. *Milkiopsis ciliata*, a native species to the UAE, had an agreement in its ranking by both the field and the image analysis techniques.

### Kruskal-Wallis test results and the effects of grazing intensities

Plant species’ cover using digital images revealed non-significant Chi-square values for Al-Maha and DDCR at P > 0.05. This reflects agreements between field and close-range photography in ranking cover of floral species. Classification within the DDCR site, however, the Chi-square was significant at P < 0.05 (Table 2). The Chi-square values were 17.6 and 20.2 for Al-Maha and DDCR; respectively. Similar results were observed for the field sampling with Chi-square significance levels at P < 0.05, relating Al-Maha and DDCR vegetation variables. Significant Kruskal-Wallis tests reflect disagreements in the median rankings of both field-based and imagery-based techniques.

Comparisons at the species level, within grazing intensities, showed significant Chi-square values (P < 0.05). The Chi-square values were 37.8 and 39.9 for Al-Maha and DDCR sites; respectively (Data not shown).

### Kruskal-Wallis test results and the effects of soil substrate

Within soil substrates, the ranking techniques did not reveal any significant Chi-square values (P > 0.05), except for gravel plains (P > 0.05). For sand dunes and sand flats, the Chi-square values were 0.64 and 1.78; respectively. These results lead us to believe that both ranking methods are in agreement when we are sampling desert areas that are dominated by either sand dunes and/or sand flats. Both substrates have been reported to constitute important soil types in the UAE desert ecosystems [25].

Comparisons at the species level, within soil substrates, showed significant Chi-square values for sand dunes and sand flats (P < 0.05). The Chi-square was 25.2 and 28.3 for the respective substrates. Field-based and image-based classification were compatible for gravel plains, as the Kruskal-Wallis test was not significant at P > 0.05

## Discussion

The present study, through the use of Pearson correlation analyses, has demonstrated that digital photography could not be used as a reliable sampling technique of floral cover at both community and species levels in desert ecosystems. Because of species cover is a key indicator of ecosystem health [20]. As most studies reported success of using digital photography only when dealing with over-all species cover in more homogeneous vegetations [16,26]. Moreover, correlation was higher for conventional methods when compared to digital photography [27]. Species level estimation of cover using pixel analysis is yet to be fine-tuned and be proven more reliable [28]. In the present study, *L. arabicum*, a locally common sand dune occupying desert species [24,25], was ranked seventh and fourth using field sampling and digital photography; respectively.

In the moderately grazed area (Al-Maha), both *H. kotschyi* and *R. epapposum* were recorded among the top six species by the two techniques in the gravel plains, but did not appear at all in the other substrate types (sand dunes and sand flats). *Rhanterium epapposum*is a very palatable species for camels; consequently, it is recovered in Al-Maha, which is moderately grazed by antelopes. In addition, *H. kotschyi* might have been introduced with the release of the antelope in the site (El-Alqamy, unpublished). This species was not recorded in the study area in an earlier floral survey; before the introduction of the wild antelope. In addition, Gallacher and Hill [29] reported a relatively lower abundance for *H. kotschyi* in a relatively more recent survey. On the other hand, all the top six species recorded in the DDCR are either unpalatable (*L. pyrotechnica* and *C. procera*) or tolerating to grazing animals (*H. salicornicum* and *C. conglomeratus*) [25].

The story was not too clear when it comes to comparing the two classification techniques within different grazing intensities. The use Kruskal-Wallis tests, however, showed that plant species cover using photos revealed non significant Chi-square values for conservatively grazed (Al-Maha) and heavily grazed (DDCR) communities. This reflected compatibility between field based and photography-based techniques in ranking floral species’ cover. For classification within the DDCR site, however, the Chi-square was revealing discrepancies in ranking using both techniques. Significant Kruskal-Wallis tests mean disagreements in the median rankings of both field-based and imagery-based techniques. Overall, image-based assessment seem to work better as plant cover increases and this is one explanation that the technique was much worst in heavily grazed areas of the study site.

Within soil substrates, the ranking techniques did not reveal any significant Chi-square values, except for gravel plains. Variations in image reflection and pixel signatures may be a reason that this substrate showed such significant values. Unfortunately, gravel plains were reported to constitute a crucial habitat preferred by grazing camels [29,30]. Careful management of these substrates have been recommended [25]. Particularly on the gravel plains, it has been reported that animals spend more time and consequently would affect seed dispersal, and hence, the distribution pattern of the different plant species [2,25]. For sand dunes and sand flats, the Chi-square values were 0.64 and 1.78; respectively. These results lead us to believe that both ranking methods are in agreement when we are sampling desert areas that are dominated by either sand dunes and/or sand flats. Both substrates have been reported to constitute important soil types in the UAE desert ecosystems [25].

At the species level, within grazing intensities, the Kruskal-Wallis tests showed significant Chi-square values, which reveals incompatibility between the two ranking techniques. Within soil substrates, the test also showed significant Chi-squares for sand dunes and sand flats. Field-based and image-based classification, however, were compatible for gravel plains, as the Kruskal-Wallis test was not significant.

Overall, field and image classification of desert ecosystems are not highly compatible at the species, soil substrate and grazing intensity levels. Pixel analysis [9], therefore, is yet to be fine-tuned and proven more reliable mainly because of the scarcity of floral cover and composition in deserts. The lack of robust results using digital photography may be attributed to plot size [22] and heterogeneity of species cover and distribution. Few studies [20] suggested that very large number of images could be a way to overcome lack of precision in estimating floral cover. Accuracy of estimation of plant cover has also been reported to be improved with the use of close range photography [31,32]. In this study an aluminum frame was suggested for repeated estimates [32] while in another trial a camera stand was constructed [28]. Further testing, at various temporal as well as spatial scales, of close range photography within desert ecosystems is required before being adopted as reliable and practical.

## Conclusion

Digital photography could not be used reliably to asses floral cover, while further testing is required to support such claim. Additionally, it is highly recommended that we use complementary techniques and procedures to digital photography when estimating floral cover at the species level in such hyper arid environments. An image-analysis based approach of floral cover at the species level and across different grazing and soil heterogeneities in desert ecosystems, has its advantages. But care is to be taken, however, during the interpretation of the results. As such approach, using digital images, is site specific.

## Methods

### Study area

The study was conducted at the Dubai Desert Conservation Reserve, United Arab Emirates. The site is made-up of two sections, where camel are freely grazing (referred to as DDCR) and a core area where strict conservation measures are in place, camel grazing is excluded, off-roading controlled and wildlife is abundant (referred to as Al-Maha area or AMR). Unfortunately, the larger Dubai Desert Conservation Reserve (DDCR) was suffering heavy grazing pressure by free ranging Camels, but off-road driving was relatively minimized while violations are still being recorded. These differences are being present for about 7–8 years since the site has been setup. Three soil types or substrates are present at both sections: sand dunes, sand flats and gravel plains.

### Approach

Field sampling procedure involved using random sampling points selected over the 3 main habitats; namely gravel plains, sand flats and sand dunes. A total of 126 plots (86 from Al-Maha and 40 from DDCR) were selected: 36 were on sand dunes (21 in the AMR and 15 in the DDCR), 28 were on sand flats (16 in the AMR and 12 in the DDCR) and 62 were on gravel plains (49 in the AMR and 13 in the DDCR) [25]. For the sand flats and sand dunes, plots were selected using a 500 m grid selected over the whole area. Circular plots were used for the sampling. Each circular plot involved sampling an area of 50m diameter. This way each plot is equivalent to 7000 *m*^2^. The percent cover for each species was measured within each sampling quadrat.

### Procedure

**Figure 1:**
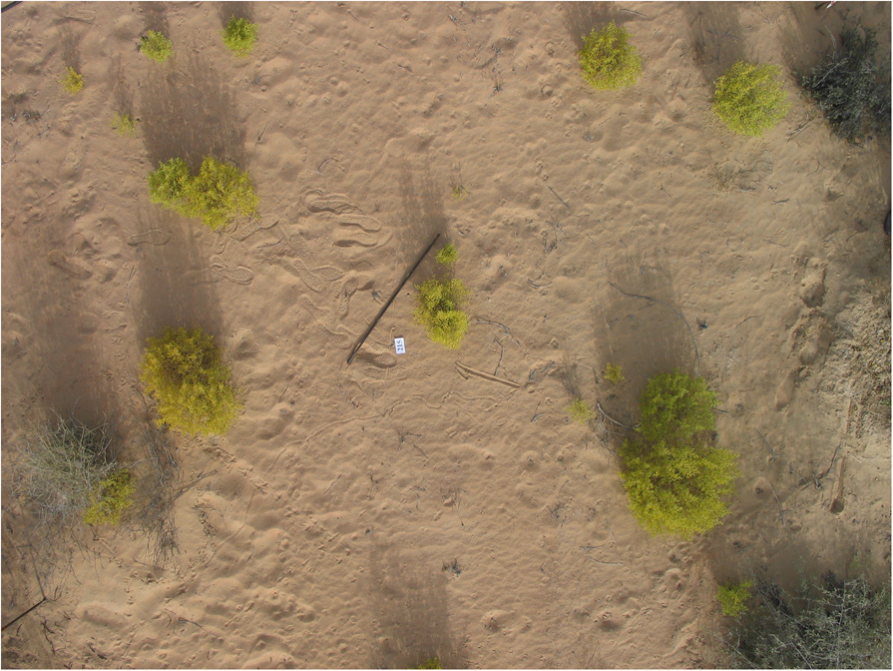
Sample vertical image for a plot assessing floral cover in the UAE desert ecosystem.

Close range photography was used to take picture of each plot from breast height of about 1.5 meters above ground using a Canon Power Shot camera with a 5-megapixel resolution. Images were within the center of the sampled area. Images taken at close range were pixel-analyzed using ERDAS imagine [33]. The procedure involved pixel analysis to quantify percent cover for each species within each of the plots. We used a supervised classification where a training group of pixels was hand-selected and applied for each species. A total of 114 images were analyzed (Sand Dunes 36, Sand flats 27 and Gravel Plains 50 images). Data from the images were compared to species cover data collected in the field. All distorted and/or off-center images were not included in the analysis. A sample field image is included in Figure 1.

Another step of the analysis involved agreements on each species’ ranking using both field-based and image-based techniques. A Pearson correlation analysis was performed to assess compatibility between the two methodologies (Table 1). Furthermore, separate Kruskal-Wallis tests were performed using SPSS [34]. One involved comparing field and photo analyses techniques as affected by grazing intensities (Al-Maha and DDCR; Table 2), while the second dealt with the effects of soil substrate (Sande dunes, Sand flats and Gravel plains) using the two sampling techniques (Table 3). A third Kruskal-Wallis test was performed for every plant species’ ranking, averaged over grazing intensities and soil substrates (Table 4). The hypothesis for the formers was that the median score of field ranking, within each grazing intensity or soil substrate, is not significantly different than the median score of image ranking. The hypothesis for the latter was that the median score of field ranking, for each species, is not significantly different than the median score of image ranking.

**Table 3:** Kruskal-Wallis test results of level of agreements between field and digital imaging and the effects of grazing intensities on estimating plant cover in a desert ecosystem

**Grazing intensities**	**Chi-Square values**	**P values**
Al-Maha	5.8	0.016
DDCR	1.5	0.216

**Table 4:** Kruskal-Wallis test results of level of agreements between field and digital imaging and the effects of soil substrates on estimating plant cover in a desert ecosystem

**Soil substrate**	**Chi-Square values**	**P values**
Sand dunes	0.64	0.423
Sand flats	1.78	0.182
Gravel plains	6.45	0.011

## Competing interests

The authors declare that they have no competing interests.

## Authors’ contributions

TSK carried out the image analysis and statistical assessment of the collected data. AAE supervised the field sampling and the vegetation identification processes. All authors read and approved the final manuscript.
